# A Parasitic Resistance-Adapted Programming Scheme for Memristor Crossbar-Based Neuromorphic Computing Systems

**DOI:** 10.3390/ma12244097

**Published:** 2019-12-08

**Authors:** Son Ngoc Truong

**Affiliations:** Faculty of Electrical and Electronics Engineering, Ho Chi Minh City University of Technology and Education, Ho Chi Minh City 70000, Vietnam; sontn@hcmute.edu.vn; Tel.: +84-93-108-5929

**Keywords:** memristor, crossbar array, neuromorphic computing, wire resistance, synaptic weight, character recognition

## Abstract

Memristor crossbar arrays without selector devices, such as complementary-metal oxide semiconductor (CMOS) devices, are a potential for realizing neuromorphic computing systems. However, wire resistance of metal wires is one of the factors that degrade the performance of memristor crossbar circuits. In this work, we propose a wire resistance modeling method and a parasitic resistance-adapted programming scheme to reduce the impact of wire resistance in a memristor crossbar-based neuromorphic computing system. The equivalent wire resistances for the cells are estimated by analyzing the crossbar circuit using the superposition theorem. For the conventional programming scheme, the connection matrix composed of the target memristance values is used for crossbar array programming. In the proposed parasitic resistance-adapted programming scheme, the connection matrix is updated before it is used for crossbar array programming to compensate the equivalent wire resistance. The updated connection matrix is obtained by subtracting the equivalent connection matrix from the original connection matrix. The circuit simulations are performed to test the proposed wire resistance modeling method and the parasitic resistance-adapted programming scheme. The simulation results showed that the discrepancy of the output voltages of the crossbar between the conventional wire resistance modeling method and the proposed wire resistance modeling method is as low as 2.9% when wire resistance varied from 0.5 to 3.0 Ω. The recognition rate of the memristor crossbar with the conventional programming scheme is 99%, 95%, 81%, and 65% when wire resistance is set to be 1.5, 2.0, 2.5, and 3.0 Ω, respectively. By contrast, the memristor crossbar with the proposed parasitic resistance-adapted programming scheme can maintain the recognition as high as 100% when wire resistance is as high as 3.0 Ω.

## 1. Introduction

**Figure 1:**
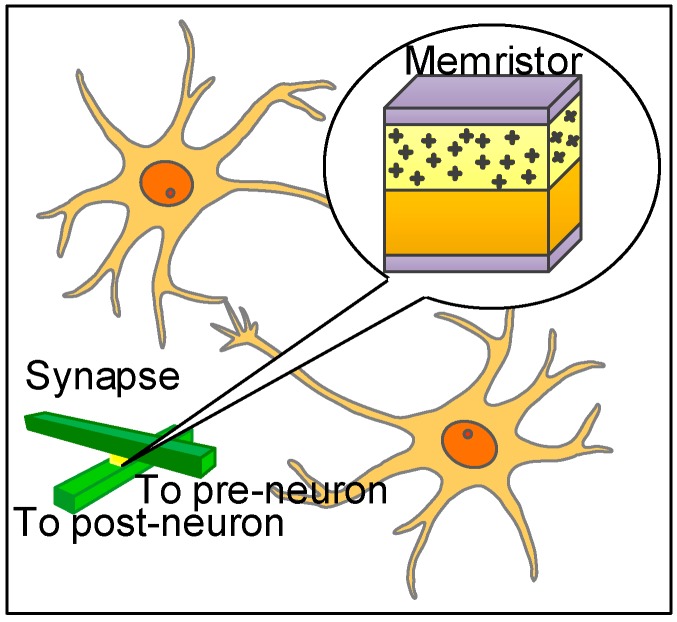
A conceptual diagram of a memristor-based synapse [11].

Neuromorphic computing was investigated by C. Mead in the late 1980s as a hardware-based approach for artificial intelligence [1]. The word “Neuromorphic” refers to an electronic circuit that is based on digital and analog components to mimic the neurobiological structures in nervous systems. Neuromorphic computing systems can be implemented on various VLSI (very-large scale integration) systems [2,3,4,5,6]. The prevailing VLSI technology today comprises mainly of CMOS (complementary-metal oxide semiconductor) devices. However, CMOS technology is approaching the end of their capabilities because scaling CMOS down faces several fundamental limiting factors stemming from electron thermal energy and quantum-mechanical tunneling [7,8]. The emerging memristive devices, termed memristors, have been considered a promising candidate for realizing the neuromorphic computing systems. Memristor was postulated by L. O. Chua in 1971 as the fourth fundamental passive circuit element and experimentally demonstrated by HP (Hewlett Packard) Labs in 2008 [9,10]. Memristors has been potentially used to implement the neuromorphic computing systems because the nonlinear relationship between magnetic flux and electric charge of memristors is very similar to the plasticity behavior of biological brain [11,12]. In biological brains, synapse is the connection between a presynaptic neuron and a postsynaptic neuron. The strength of a synapse is represented by a synaptic weight. According to the neuron activities including excitatory and inhibitory, synaptic weights can be positive or negative [13,14]. Synapses can be modeled by memristors as shown in Figure 1 [11]. The synaptic weight is represented by the conductance of memristor, which can increase or decrease according to the current flowing through the device.

A memristor crossbar array is a fully connected mesh of perpendicular wires, in which any two crossing wires are connected by a memristor [15]. Neuromorphic computing systems employing crossbar architecture of memristors have gained more advantages in terms of the flexibility, power consumption, cost, and area [16,17,18,19,20,21,22,23]. Miao Hu et al. proposed a crossbar architecture of synaptic array composing of a plus and minus crossbar arrays representing plus- and minus-polarity connection matrices for analog neuromorphic computing [20]. To reduce the area and power consumption, S. N. Truong proposed a new memristor crossbar architecture, which is composed of a single memristor array and a constant-term circuit [21]. The proposed architecture can reduce the power consumption by 48% and the area by 50% [21]. The memristor crossbar has also applied to the applications of speech recognition and image recognition [22,23].

In a memristor crossbar array, some amount of voltage drop can be caused by parasitic resistance, also known as wire resistance along the row and the column lines [19,24,25,26,27,28]. Hereinafter “wire resistance” and “parasitic resistance” are used interchangeably. The impact of wire resistance becomes inevitable when the array size increases [22]. To mitigate the impact of wire resistance, several interesting schemes were proposed [25,26,27,28]. A design methodology has been proposed to reduce the impact of wire resistance in a one-selector-one resistive device (1S1R) crossbar array [27]. The proposed design methodology seems to be complicated since the physical specification of the devices must be considered [27]. Another approach to deal with the wire resistance is to use a dynamic reference scheme [25]. The read operation is performed with two steps associated with a special reading circuit. [25]. These proposed schemes are effective when they are applied to a memristor crossbar array, in which memristors are used as binary switches between two distinct high and low resistance states (HRS (High Resistance State) and LRS (Low Resistance State)). These solutions are mainly based on the additional techniques or circuits to compensate the variation of reading voltage caused by wire resistance. To the best of our knowledge, there is a lack of the techniques that can be applied to the programming process of crossbar circuit to lessen the impact of wire resistance in the inference process. In this work, we propose a parasitic resistance-adapted programming scheme for memristor crossbar-based neuromorphic computing systems, in which memristors are used as analog connections. An equivalent wire resistance is proposed for modeling wire resistance in crossbar circuit. The proposed equivalent wire resistance matrix is used to compensate wire resistance during the programming process. As the result, the impact of wire resistance in inference process is reduced significantly.

## 2. Materials and Methods 

**Figure 2:**
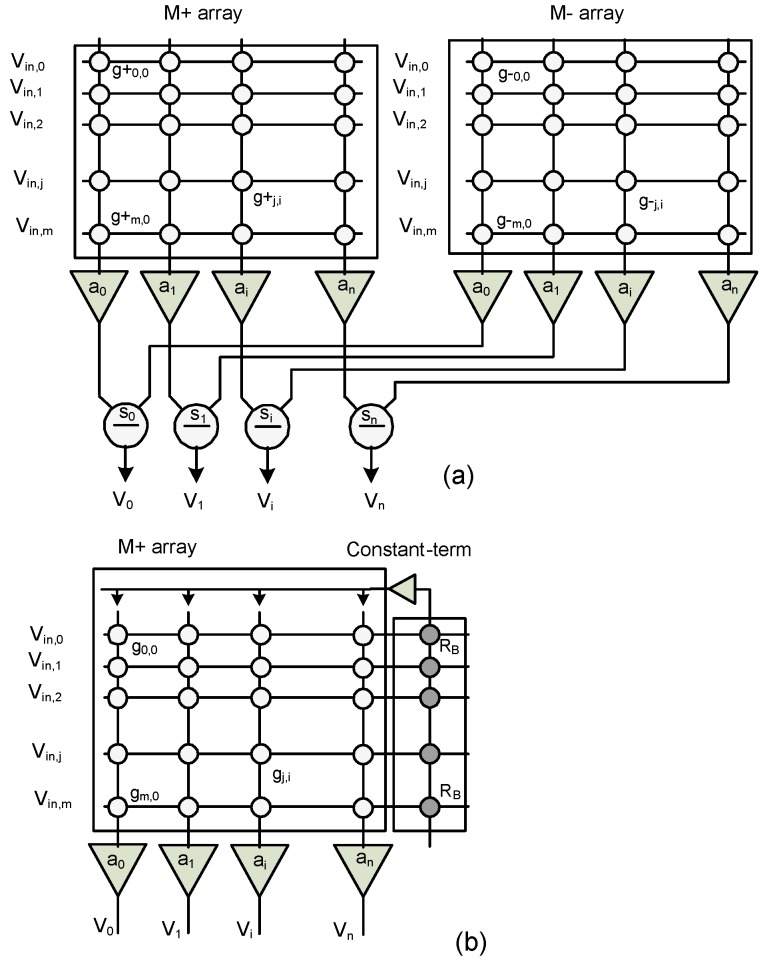
(**a**) The conceptual diagram of two crossbar arrays for implementing plus- and minus-polarity connection matrices [20] and (**b**) the optimized crossbar architecture, which employs only one memristor crossbar and a constant-term circuit for realizing negative and positive synaptic weights [21].

In neuromorphic computing systems, the synaptic weights obtained from the training process are either positive or negative according to they are excitatory synapses or inhibitory synapses [13,14]. The signal passing through these synaptic connections can be strengthened or weakened. When modeling biological synapses using memristors, it should be guaranteed that the synaptic weights could be negative values or positive values, consistent with the inhibitory or excitatory synapses. For doing this, the crossbar architecture with two memristor crossbar arrays for plus and minus connection matrices was proposed [20]. Figure 2a shows a conceptual diagram of crossbar architecture of an analog neuromorphic computing system [20]. Here plus-polarity and minus-polarity connection matrices are utilized to implement the synaptic array, in which synaptic weights can be programmed to be negative or positive. The circles in Figure 2a represent the memristors that connect the inputs and the columns. a_0_ to a_n_ are additions, and s_0_ to s_n_ are subtractions that produce the output voltages from *V*_0_ to *V*_n_. *g*^+^_0,0_ is the memristor’s conductance value of the crossing point between the first row and the first column in M^+^ array. Similarly, *g^−^*_0,0_ is the memristor’s conductance in M- array, as shown in Figure 2a. The output voltage for the *i*th column can be calculated as

(1) $$\begin{array}{l} V_{i}=\sum_{j=0}^{m}V_{in,j}g^{+}{}_{j,i}-\sum_{j=0}^{m}V_{in,j}g^{-}{}_{j,i}\\ V_{i}=\sum_{j=0}^{m}V_{in,j}w_{j,i}\\ Here,\;w_{j,i}=(g^{+}{}_{j,i}-g^{-}{}_{j,i}) \end{array}$$

In Equation (1), the output voltage is a summation of inputs, which are weighted by the corresponding weights, *w_j,i_*. The synaptic weight, *w_j,i_*, is decided by the difference of two conductance values of memristors in two arrays; *g^+^_j,i_* in the M^+^ array, and *g^−^_j,i_* in M^−^ array. To reduce the power consumption and area, S. N. Truong proposed a new crossbar architecture, which employed only one crossbar array and a constant-term circuit [21]. The proposed crossbar architecture is conceptually shown in Figure 2b. There is only one memristor crossbar array instead of two memristor crossbar arrays for representing the signed synaptic array. The negative synaptic weight is generated using an additional column, which connects to the inputs through *R*_B_s, as shown in Figure 2b. Here, a constant-term circuit is used to replace a crossbar array without changing the functionality of the crossbar circuit [21].

**Figure 3:**
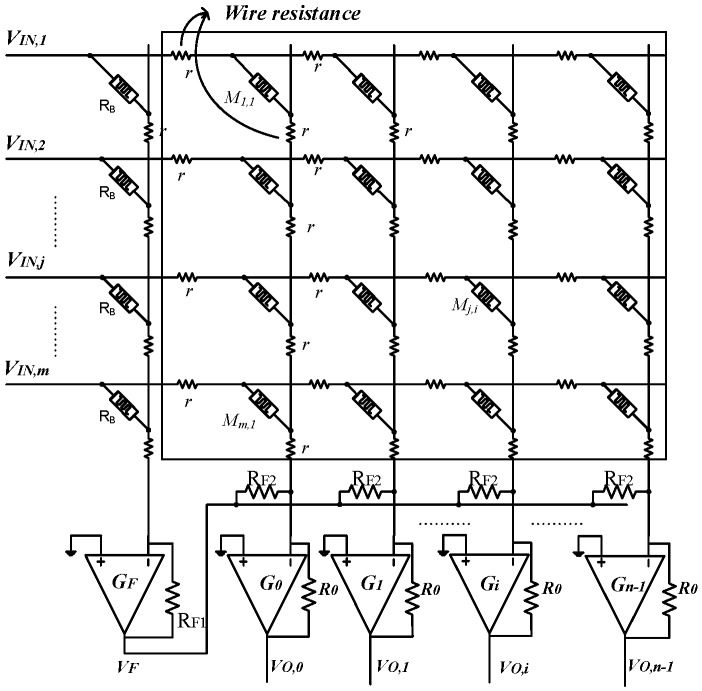
The schematic of memristor-based neuromorphic computing circuit with the presence of wire resistance. Wire resistance is modeled by small-value resistors on vertical lines and horizontal lines.

In previous works, memristor crossbar circuits are simulated with ignoring the presence of wire resistance. However, the impact wire resistance in crossbar is inevitable. It becomes more serious as the array size increases [25]. Wire resistance is modeled by small-value resistors lying on the vertical lines and the horizontal lines, as shown in Figure 3. In Figure 3, if wire resistance is omitted, the output voltage of the *i*th column is calculated by Equation (2) [21].

(2) $$\begin{array}{l} V_{O,i}=\sum_{j=0}^{m}V_{in,j}w_{j,i}\\ where,\;w_{j,i}=R_{0}(\frac{1}{R_{B}}-\frac{1}{M_{j,i}}) \end{array}$$

Equation (2) is used for calculating the output voltage of the *i*th column. The output of each column is a summation of the weighted inputs, hence each column works as a perceptron neuron. In Equation (2), *M_j,i_* is the memristance value of the crossing point between the *j*th row and *i*th column. R_B_ is a constant, the synaptic weight, w*_j,i_*, can be decided to be either negative or positive by adjusting the memristance, *M_j,i_*.

**Figure 4:**
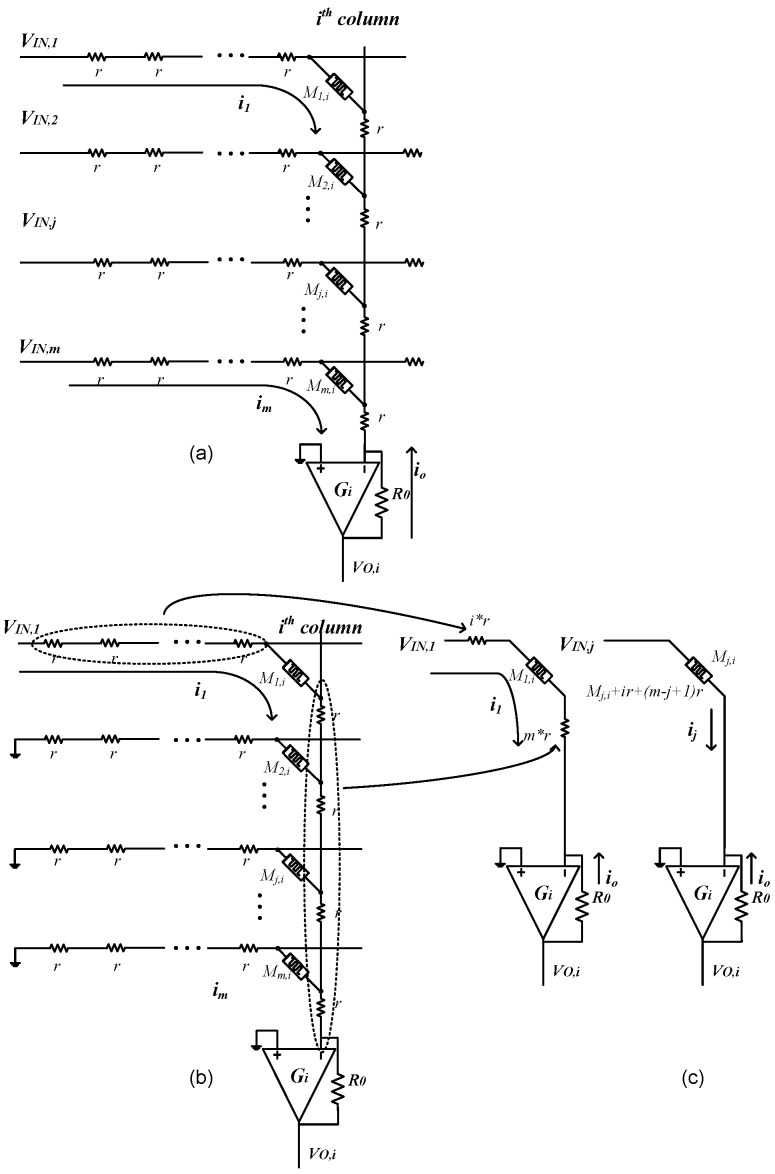
Analyzing the crossbar circuit using superposition method. (**a**) The schematic of the *i*th column with the presence of wire resistance; (**b**) analyzing the circuit using superposition method, and (**c**) the equivalent wire resistance for the cell M_j,i_.

If wire resistance is not omitted, it can be modeled by small-value resistors along vertical and horizontal lines as shown in Figure 3. The *i*th column of crossbar is separated and shown in Figure 4. The output voltage of the *i*th column is calculated by applying Ohm’s law and the Kirchhoff’s current law to the node of V^−^ of the Op-amp, as presented in Equation (3).

(3) $$\begin{array}{l} V_{o,i}=R_{0}i_{0}\\ where\;\;i_{0}+\sum_{j=1}^{m}i_{j}=0 \end{array}$$

To analyze the circuit in Figure 3, we can use the well-known superposition theorem. In particular, we isolate the circuit row by row as shown in Figure 4a. When we calculate the current for the *j*th row, we can assume that the inputs for other rows are zero, as shown in Figure 4b. Since the value of the resistor, r, is very small compared to the memristance values, the circuit in Figure 4b can be approximated by using the equivalent circuit, as illustrated in Figure 4c. In Figure 4c, the resistors, which the current i_1_ passes through, can be approximately represented by an equivalent resistor *R*_1,*i*_:

(4) $$R_{1,i}=ir+mr$$

where *R*_1,*i*_ is an equivalent wire resistance for cell *M*_1,*i*_. In general, we can approximate the wire resistance for the cell *M_j,i_* as follows

(5) $$R_{j,i}=ir+(m-j+1)r$$

where, *m* is the number of rows in the crossbar circuit. *r* is wire resistance value.

**Figure 5:**
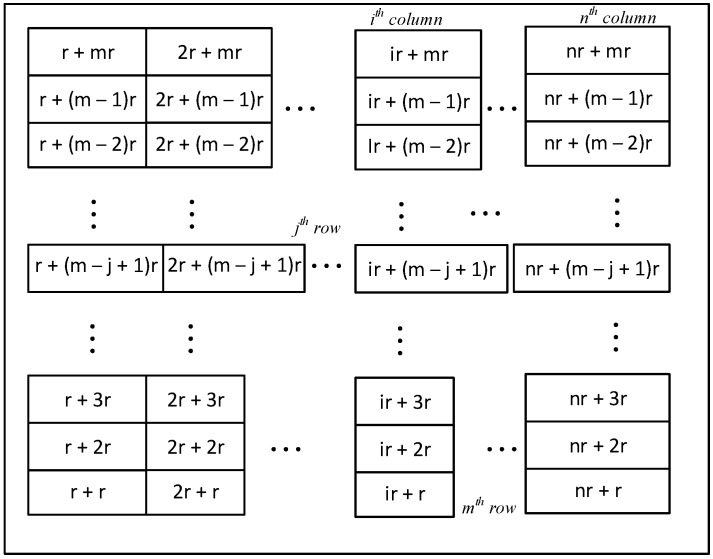
The proposed equivalent wire resistance matrix for modeling wire resistance in an m × n crossbar array. Here r is the value of wire resistance, m is the number of rows, and *n* is the number of columns.

In this work, we proposed a wire resistance modeling method by using the proposed an equivalent wire resistance matrix for an m × n crossbar array, as illustrated in Figure 5. The elements in the proposed matrix are the equivalent resistance values of wire resistance on vertical line and horizontal line, which are calculated by Equation (5) for the corresponding cells. 

**Figure 6:**
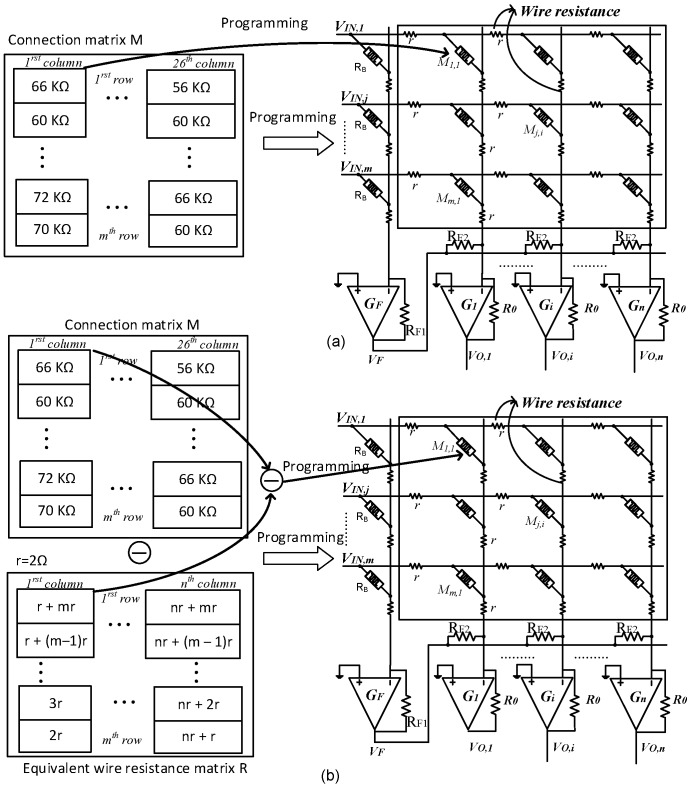
(**a**) The conventional programming scheme, in which the memristance values in connection matrix are used to program the corresponding cells in crossbar array and (**b**) the proposed parasitic resistance-adapted programming scheme, where the value of connection matrix is updated by subtracting the proposed equivalent wire resistance matrix from the original connection matrix. The updated connection matrix is then used to program the crossbar array. *R* is the proposed equivalent wire resistance matrix for an m × n crossbar array. *r* is the value of wire resistance, m is the number of rows, and n is the number of columns.

The proposed equivalent wire resistance matrix was used to compensate the impact of wire resistance in crossbar array by adjusting the connection matrix according to the proposed equivalent wire resistance matrix. In particular, we proposed a parasitic resistance-adapted programming scheme to compensate wire resistance for a memristor crossbar-neuromorphic computing. The proposed scheme is conceptually shown in Figure 6b. Figure 6a shows a conventional programming scheme for a crossbar circuit. The synaptic weights that were obtained from the training process were converted to the values of memristance using Equation (2). The memristance values of the cells in crossbar form a connection matrix M as presented in Figure 6. For the conventional programming scheme, the cells in the crossbar array were programmed to the target values presented in the connection matrix M. Wire resistance was not considered during programming process and inference phase. To consider the presence of wire resistance, the connection matrix was updated before it is used to program the crossbar array. Specifically, the target memristance matrix was obtained by subtracting the proposed equivalent wire resistance matrix from the original connection matrix, as conceptually shown in Figure 6b. By updating the connection matrix with the proposed equivalent wire resistance matrix, wire resistance was compensated in the inference phase. The connection matrix is updated using the Equation (6)

(6) $$\begin{array}{l} M_{j,i}=M_{j,i}-R_{j,i}\\ =M_{j,i}-ir+(m-j+1)r \end{array}$$

where, *M_j,i_* is memristance of the cell between the *j*th row the *i*th column. In the conventional programming scheme, the cell *M_j,i_* is programmed to have the memristance of *M_j,i_*. In the proposed programming scheme, the cell *M_j,i_* is programmed to have the memristance of *M_j,i_* − *ir* + (*m* − *j* + 1)*r*, where the amount of *ir* + (*m* − *j* + 1)r represents the equivalent wire resistance for the cell *M_j,i_*. By doing this, wire resistance is compensated in the inference phase. 

## 3. Results

**Figure 7:**
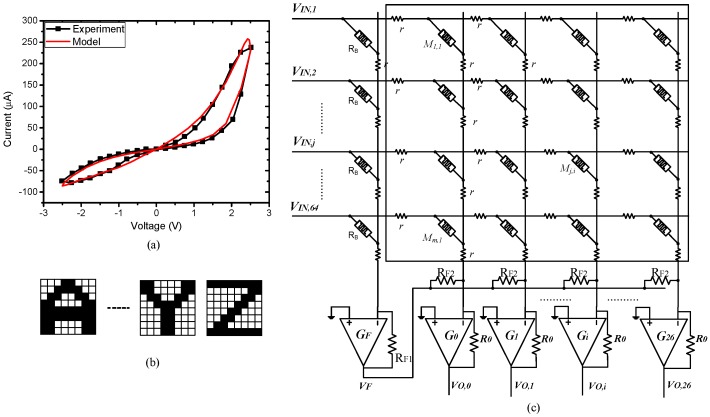
(**a**) The memristor’s current–voltage characteristic measured from the real device and the memristor’s behavior model; (**b**) the eight × eight images of characters used to test the proposed equivalent wire resistance modeling method and the parasitic resistance-adapted programming scheme; and (**c**) the schematic of crossbar circuit for the application of character recognition.

The circuit simulations were performed to verify the proposed wire resistance modeling method and the parasitic resistance-adapted programming scheme for a memristor crossbar-based neuromorphic computing system. The simulations were performed using the SPECTRE circuit simulation provided by Cadence Design Systems Inc. [29]. Memristors were modeled using Verilog-A and CMOS technology is given by SAMSUNG 0.13 mm process technology [30,31]. Figure 7a shows a hysteresis behavior of a real memristor based on the film structure of Pt/LaAlO_3_/Nb-doped SrTiO_3_ stacked layer and a memristor model that can be used to describe various memristive behaviors [30,31]. The memristor model and parameters are presented in [30]. The crossbar circuit was used for the application of character recognition. Figure 7b shows eight × eight images of characters used in these simulations. Each character was composed of 64 black-and-white pixels. The crossbar circuit was schematically shown in Figure 7c for recognition of the characters from “A” to “Z”. To recognize 26 characters, the memristor crossbar was composed of 26 columns and a constant-term of R_B_ as depicted in Figure 7c. The constant-term column connected to all inputs through R_B_ to generate the negative voltage as mentioned in the previous work [21]. The crossbar had 26 columns corresponding to 26 perceptron neurons for recognizing 26 characters from “A” to “Z”. For example, the first column is trained to be activated with the input character “A” and the 26th column is trained to be activated with the input character “Z” [21]. Wire resistance was modeled by small-value resistors along vertical and horizontal lines, as shown in Figure 7c. Here R_B_ and R_0_ were set to be 60 KΩ and 200 KΩ respectively. *R*_F1_ should be equal to *R*_F2_ as mentioned in previous work [21]. 

**Figure 8:**
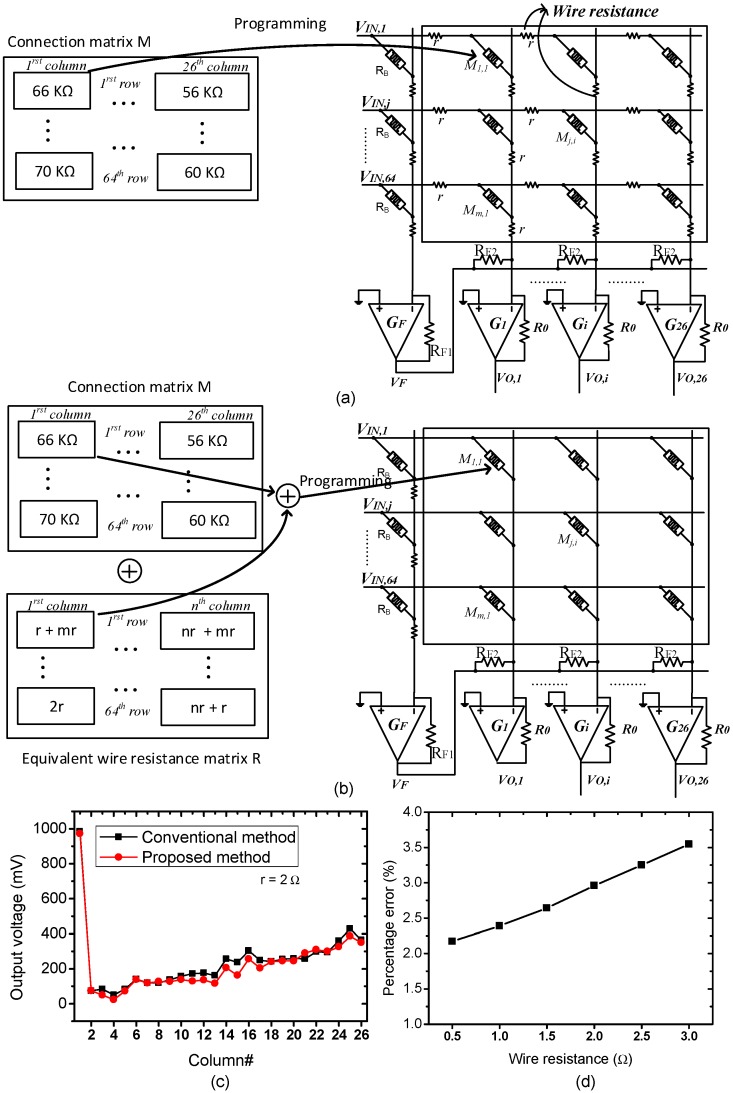
(**a**) The conventional method for the crossbar circuit simulation with taking the presence of wire resistance into account. Here wire resistance is modeled by small-value resistors along the vertical and horizontal lines; (**b**) the proposed method with equivalent wire resistance for the crossbar circuit simulation with considering the presence of wire resistance. Here, the small-value resistors are not present in the crossbar circuit, the connection matrix is updated by adding the equivalent wire resistance matrix to the connection matrix instead; (**c**) the output voltages of 26 columns for the input character “A” and (**d**) the percentage error with varying wire resistance from 0.5 to 3.0 Ω.

The proposed wire resistance modeling method using equivalent wire resistance matrix was verified by the simulation that was set up as presented in Figure 8a,b. The synaptic weights obtained from the training process were converted to the memristance values in connection matrix using Equations (2) and (6). For the conventional method, wire resistance was modeled by small-value resistors along vertical and horizontal lines, as shown in Figure 8a. The crossbar was programmed to the target memristance values presented in the connection matrix using the V_DD_/3 write scheme [32]. For the proposed method, we calculated the equivalent wire resistance matrix as shown in Figure 5. The small-values resistors were not present in the crossbar circuit, the value of equivalent wire resistance matrix was added to the connection matrix instead, as conceptually shown in Figure 8b. In other words, the connection matrix was updated by adding corresponding elements of the connection matrix and the proposed equivalent wire resistance matrix. The crossbar was then programmed to the target memristance values presented in the updated connection matrix using V_DD_/3 write scheme. In Figure 8c, the output voltages of 26 columns for recognizing 26 characters were measured when the vector of character “A” was applied to the inputs. Among the 26 columns, only the first column produced high voltage for recognizing character “A”. When wire resistance was set to be 2.0 Ω, the voltage drop on wire resistance made the output voltages of columns increase, as shown in Figure 8c [33]. Since the voltage drop on wire resistance depends on the length of metal line, the column close to the first column had less change of voltage whereas the column far from the first column had much change of voltage, as demonstrated in Figure 8c [33]. The result obtained from the conventional method is represented by the square symbols and that one obtained from the proposed method with equivalent wire resistance matrix is represented by the round symbols. The discrepancy between the two methods was as low as 3%. 

In Figure 8d, we calculated the percentage error, which is defined as the difference of the output voltages between the conventional wire resistance modeling method in Figure 8a and the proposed wire resistance modeling method in Figure 8b, in which wire resistance was modeled using the proposed equivalent wire resistance matrix. In these simulations, wire resistance was varied from 0.5 to 3.0 Ω. This range of wire resistance is commonly used and obtained from the International Technology Roadmap for Semiconductors [24,25,34,35,36,37]. When wire resistance was set to be 0.5 Ω, the percentage error was as low as 2.2%. The percentage error increased slightly when wire resistance increased, as shown in Figure 8d. On average, the discrepancy between the two methods was as low as 2.9%. The simulation results indicate that wire resistance in crossbar circuit could be modeled using the proposed equivalent wire resistance matrix, which is presented in Figure 5.

**Figure 9:**
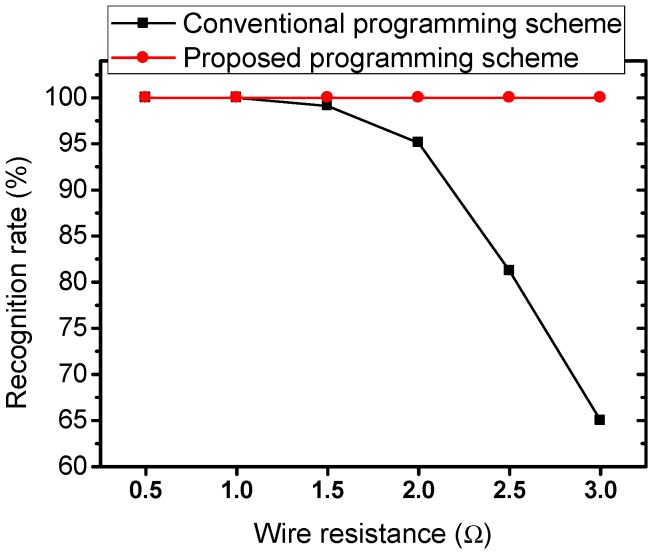
The comparison of recognition rate between the conventional programming scheme and the proposed parasitic resistance-adapted programming scheme when wire resistance is varied from 0.5 to 3.0 Ω.

Figure 9 shows the comparison of the recognition rate of memristor crossbar array between the conventional programming scheme and the proposed parasitic resistance-adapted programming scheme for recognizing 26 characters when wire resistance was varied from 0.5 to 3.0 Ω. For the conventional programming scheme, the connection matrix obtained from the training process of memristor crossbar for recognition of 26 characters was used for the crossbar array programming. In the proposed parasitic resistance-adapted programming scheme, the connection matrix was updated by subtracting the proposed equivalent wire resistance matrix from the original connection matrix. The updated connection matrix was then used for the crossbar array programming. The recognition rate of the memristor crossbar with using conventional programming scheme declined dramatically when wire resistance increased. This was due to the fact that the synaptic weight is a nonlinear function of memristance as presented in Equation (2), the change of memristance caused by wire resistance makes the synaptic weight change remarkably. As a result, the recognition rate was degraded dramatically. In particular, the recognition rate of the memristor crossbar with using the conventional programming scheme was 99%, 95%, 81%, and 65% when the wire resistance was set to be 1.5, 2.0, 2.5, and 3.0 Ω, respectively, as indicated in Figure 9. The presence of wire resistance causes the output voltage increased as mathematically analyzed and experimentally demonstrated in previous work [33]. The last column had the large variation of output voltage caused by wire resistance [33]. Therefore, the increase of wire resistance caused the recognition rate to decrease significantly, as the shown in Figure 9. By contrast, the memristor crossbar with using the proposed parasitic resistance-adapted programming scheme could maintain the recognition as high as 100% when wire resistance was as high as 3.0 Ω. This was because the value of memristance in connection matrix was updated by subtracting the equivalent wire resistance matrix from the original connection matrix. By doing this, the wire resistance in crossbar circuit was compensated.

Wire resistance degraded the performance of crossbar circuit dramatically. In this work, we tried to mitigate the impact of wire resistance by compensating wire resistance. It was done by adjusting the memristance values before they were used to program the crossbar array. In particular, the connection matrix was updated by subtracting the equivalent wire resistance matrix from the original connection matrix. By doing this, no additional circuits or components were required. The proposed parasitic resistance-adapted programming scheme was effective for memristor crossbar-based neuromorphic computing systems.

## 4. Conclusions

Wire resistance is one of the factors that degrade the performance of the crossbar circuits significantly. In this work, we proposed a parasitic resistance-adapted programming scheme to mitigate the impact of wire resistance in memristor crossbar array. Firstly, a wire resistance modeling method using equivalent wire resistance matrix was proposed. The equivalent wire resistance matrix was achieved by analysis the crossbar circuit using the superposition method. The connection matrix was updated before it was used as a target for memristor crossbar programming. The updated connection matrix was obtained by subtracting the proposed equivalent wire resistance matrix from the original connection matrix. The circuit simulations were performed to verify the proposed wire resistance modeling method and the parasitic resistance-adapted programming scheme. The simulation results showed that the discrepancy of the output voltages of the crossbar circuit between the conventional wire resistance modeling method and the proposed wire resistance modeling method was as low as 2.9% when wire resistance varied from 0.5 to 3.0 Ω. The recognition rate of the memristor crossbar with conventional programming scheme was 99%, 95%, 81%, and 65% when wire resistance was set to be 1.5, 2.0, 2.5, and 3.0 Ω, respectively. By contrast, the memristor crossbar with the proposed parasitic resistance-adapted programming scheme could maintain the recognition as high as 100% when wire resistance was as high as 3.0 Ω.
